# 509 Why do elderly burn patients die? Analysis of early versus delayed ICU deaths, from 2014-2021

**DOI:** 10.1093/jbcr/irac012.140

**Published:** 2022-03-23

**Authors:** Emily H Werthman, Jessica Ballou, Tomer Lagziel, Joshua S Yoon, Arya A Akhavan, Feras Shamoun, Julie Caffrey, Charles S Hultman

**Affiliations:** Johns Hopkins Bayview Medical Center, Lutherville, Maryland; Johns Hopkins, Baltimore, Maryland; Johns Hopkins University School of Medicine / Sackler School of Medicine, New-York Program, Tel-Aviv University, Rockville, Maryland; R. Adams Cowley Shock Trauma Center, Division of Plastic Reconstructive and Maxillofacial Surgery, Herndon, Virginia; Johns Hopkins Hospital, North York, Ontario; Johns Hopkins University School of Medicine, 347 Morningside Ave, Ontario; Johns Hopkins, Baltimore, Maryland; Johns Hopkins University School of Medicine, Baltimore, Maryland

## Abstract

**Introduction:**

Despite continued improvements in critical care, nutrition, and surgical technique, elderly patients with burn injury remain a vulnerable population, with increased mortality. The purpose of this study was to compare early versus late deaths in elderly burn patients admitted to the intensive care unit, to identify potential interventions that might improve survival.

**Methods:**

We conducted a retrospective review of elderly patients (age >=60 years), who were admitted to an urban burn center ICU, with thermal and/or inhalation injury, over an 8-year period. Data were extracted from a prospectively maintained registry and verified through our electronic medical record. Patients who died less than 1 week after admission were compared with those who died after the first week. Univariate analysis was performed by 2-tailed Student’s T test and chi-square, with statistical significance assigned to p values < 0.05.

**Results:**

From 2014-2021, we admitted 1322 patients to the burn ICU for thermal and/or inhalation injury. Mortality was 9.4% for patients >= 60 years of age, compared to 2.0% for patients < 60 (p< 0.001). The elderly patients who succumbed to their injury had a mean age of 75.3 years, TBSA 27.7%, modified Baux score of 111.3, and survival of 13.8 days. We observed a bimodal distribution of deaths, peaking on the first day after injury, and in the third week after admission, the most common cause of which, for both groups, was multisystem organ failure. Compared to the delayed deaths (n=21), patients who died within the first week (n=16) had an increased incidence of inhalation injury, a higher modified Baux score but similar age and TBSA, and lower baseline comorbidities and complications (TABLE).

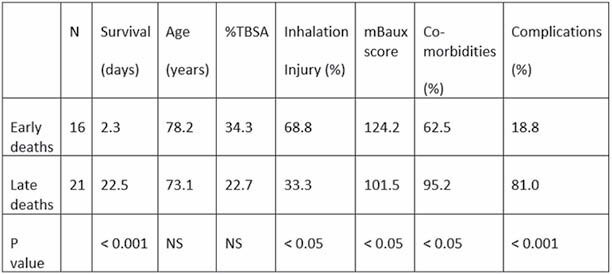

**Conclusions:**

Presence of inhalation injury and high modified Baux score, not necessarily age or %TBSA, was associated with early mortality in the elderly, after burn injury. Older patients who survive their initial resuscitation often succumb to complications related to baseline comorbidities. Improved management of these comorbidities, via the active involvement of geriatric medicine and palliative care, represents an opportunity to increase survival.

